# Dr. Wilson, on Digestion

**Published:** 1803-10-01

**Authors:** A. Philips Wilson

**Affiliations:** Worcester


### *To the Editors of the Medical and Physical Journal.*

Gentlemen,

HAVING observed in the Medical and Physical Journal, for last month, the paper of M. Dumas on the cause of hunger and thirst, I am induced to transmit to you the following observations.

A person

A person was seized, after a long confinement, with great anxiety, debility, and complete loss of appetite, which lasted for about four days, accompanied with thirst, and a failure of the saliva. During the second night, having sucked an orange, in order to remove the disagreeable dryness occasioned by the want of the saliva, he next day felt nausea and oppression referred to the stomach, which induced him to empty it, by irritating the fauces, about eight hours after the orange juice had been taken. I was much surprized to find it after that stay on the stomach, unaltered and unmixed with any other substance.

It would appear from this case, that the failure of the appetite for food is connected with a want of the gastric fluid. It was evident that there was nothing in the stomach but the orange juice, which had undergone no change; doubtless, because there was no gastric fluid present.

We must infer from the experiments of Spalanzani, Dr. Stevens, and others, that the gastric fluid is the means of effecting that change which the food undergoes in the stomach; and does it not seem probable, that the sensation of hunger may arise from the action of this fluid on the stomach, and that the absence of the gastric fluid is indicated by a want of appetite, a provision of nature, which prevents our eating at a time when no digestion can take place, by which we should only excite repeated vomiting, without receiving nourishment?

If these observations are just, we may, it would seem, by emptying the stomach of its gastric fluid, at will destroy the appetite; but it is difficult to empty the stomach entirely of the gastric fluid, both because it is difficult to empty it entirely of any of its contents, and because the very act of vomiting, excites it to pour out a fresh quantity. That the appetite, however, can be nearly destroyed, and the sensation of hunger almost entirely taken away, by removing the gastric fluid from the stomach, appears from the following experiment.

A person, in good health, having fasted for seventeen hours, and still farther increased the appetite by exercise, instead of satisfying it, by means of warm water, repeatedly excited vomiting.

The water returned clear, and only mixed with a ropy transparent fluid, such as the gastric fluid is described by Spalanzani, or as I have myself procured it from the crow, so that there had been nothing in the stomach but the water and the gastric fluid, which, like the water, was without without taste, smell, or colour. By this operation the hunger, which immediately before was urgent, was wholly removed, and rather a disgust to food produced, which was sensibly felt on seeing others eat. A small quantity of food satiated even to sickness, which continued for some hours, attended with a sense of oppression referred to the stomach.

Do not these observations seem to warrant the opinion, that the presence of the gastric fluid in the stomach, without such substances as are fit for combining with it, and thus destroying its activity, is a principal cause of death from hunger, for anorexia we have seen is occasioned by the evacuation of the gastric fluid, and we know how long people labouring under anorexia, however excited, will live without food?

The speedy acidity which took place in this experiment is remarkable. Although the stomach, as appears from the state of what was thrown up, was free from every fermenting substance, yet the food which was taken (bread and milk) acquired acidity in a quarter of an hour, indicated by acid eructations.

Does it not appear from this experiment, I may ask by the bye, that a diminution of the due quantity of gastric fluid is at least one cause of dyspepsia? Is it not probable that in such, perhaps in all cases of dyspepsia, the symptoms may be mitigated, perhaps removed, by supplying dyspeptics with the gastric fluid of those animals whose food is most similar to that of man? This I was led, from much consideration, to propose in a Treatise published eleven years ago. In a Thesis published at Edinburgh several years after by Dr. Scot, it is observed, that an Italian physician finding every thing else fail in a dyspeptic case, had recourse to the gastric fluid of brutes, which proved successful.

To return from this digression, with respect to affections of the mind removing the calls of hunger, do they not occasionally abstract our attention from all other sensations? I believe the appetite will always be found to return on the tranquillity of the mind being restored, except in debilitated habits, where the power of the gastric fluid is much impaired. The want of appetite in fevers, where the supply of the gastric fluid, as well as of other secreted fluids, fails, farther strengthens the foregoing observations.

With regard to thirst, it seems to attend all cases of diminished secretion in the alimentary canal, whether there be be a deficiency of watery fluid in the blood or not. There is no such deficiency in the commencement of fevers, and in dyspepsia.

I am, &c.

A. PHILIPS WILSON.

*Worcester, Aug.* 14, 1803.

---

